# Encourage Playing with Data and Discourage Questionable Reporting Practices

**DOI:** 10.1007/s11336-015-9445-1

**Published:** 2015-03-28

**Authors:** Daniel H. J. Wigboldus, Ron Dotsch

**Affiliations:** 1 Behavioural Science Institute, Radboud University Nijmegen, P.O. Box 9104, 6500 HE Nijmegen, The Netherlands; 2 Department of Psychology, Utrecht University, Utrecht, The Netherlands

Not so long ago, in the early days of experimental psychology, researchers performed their data analyses by hand (or paid people to do these calculations for them). No wonder, they took ample time beforehand to think through which analyses to perform (and especially which not). Exploring data was not really an option. The $t$ value or $F$ value that resulted from the calculations provided a more or less definitive answer. H0 could be rejected or not.

During the past decades, we have quickly moved from punch cards, via expensive mainframes, to relatively cheap but powerful personal computers that are fulltime available to individual researchers and students. Nowadays, SPSS and other user-friendly statistical packages make it easy to run all types of analyses, which encourage analyzing your data in every possible way. It is stunningly easy to (re-)run analyses with or without a specific group of participants, with or without an additional between-subject condition, with or without an extra variable in a scale. Indeed, it may be a waste of resources to leave a dataset partly unexplored when SPSS offers all the tools to do so. In many research labs, analyzing results has shifted from spending days to do confirmatory analyses by hand, to spending days engaging in all kinds of data analyses to see "what’s in the data.” Is this a bad thing? We do not think so, necessarily. Given that you know what you are doing in SPSS, you can learn a lot from exploring a dataset. However, there clearly are risks (see Simmons, Nelson & Simonsohn, 2011), which we will revisit later.

This contribution is a commentary on "Playing with Data—Or How to Discourage Questionable Research Practices and Stimulate Researchers to Do Things Right” by Sijtsma (2015). In his paper, Klaas Sijtsma makes the important point that prevention of questionable research practices may be more important than detection. He suggests two policy measures. First, make research data and research materials publicly available. Second, encourage researchers to consult methodologists or statisticians for help and a second opinion.

In this commentary, we will not reiterate the points made by Klaas Sijtsma. We support his analysis and his policy recommendations. Making research data and research materials publicly available and consulting methodologists and statisticians will help in increasing the transparency of the data analysis phase of the empirical research cycle within psychology. Currently, in too many cases, this phase is the exclusive domain of (small groups of) individual researchers and their personal computers. However, making the data analysis phase transparent to others will not prevent scientific fraud. A person who willingly aims to fool others always will be able to do so, also when sharing (well-faked) data. Nevertheless, it will help in reducing the errors researchers may make unwittingly due to incorrect methodological decisions and the incorrect use of statistical methods (see Sijtsma).

The current contribution focuses on what we think is quickly becoming a misnomer in our field, namely the use of the phrase "questionable research practices” for certain types of practices when analyzing and reporting data. We suggest to replace this with the, in our view, more accurate phrase "questionable reporting practices,” in relation to most potentially problematic data analysis strategies. The abbreviation may remain the same, namely QRPs. However, the meaning and use of the phrase will be more positive and more encouraging to junior and senior researchers, and will do more justice to the important distinction between confirmation and exploration when analyzing data and reporting results (see Wagenmakers, 2012).

## Questionable Research Practices

We were first introduced to the phrase "Questionable Research Practices” during a conference presentation by Leslie John (see, John, Loewenstein & Prelec, 2012). In a survey among over 2000 psychologists, John et al. measured the prevalence of what they called questionable research practices. In their paper, these practices are not explicitly defined, but are described as a "gray area of acceptable practice” (p. 524, 531). The examples they used to measure the prevalence of questionable research practices are listed in Table 1.

**Table 1:** Questionable Research Practices as measured by John et al. (2012).

1. In a paper, failing to report all of a study’s dependent measures
2. Deciding whether to collect more data after looking to see whether the results were significant
3. In a paper, failing to report all of a study’s conditions
4. Stopping collecting data earlier than planned because one found the result that one had been looking for
5. In a paper, ’Rounding off’ a *p* value (e.g., reporting that a *p* value of .054 is less than .05)
6. In a paper, selectively reporting studies that ’worked’
7. Deciding whether to exclude data after looking at the impact of doing so on the results
8. In a paper, reporting an unexpected finding as having been predicted from the start
9. In a paper, claiming that results are unaffected by demographic variables (e.g., gender) when one is actually unsure (or knows that they do)
10. Falsifying data

Looking at the prevalence rates of the practices reported by John et al. (2012), one might observe there to be substantial variation in "how gray” or (un)accepted by the field each of these research practices is. For instance, failing to report all of the study’s dependent measures seems to occur much more often than falsifying data. It should be noted that the prevalence rates reported by John et al. have been debated by others.^1^ For instance, recently, Fiedler and Schwarz (2014) argued that the high estimates of QRP prevalence reported by John et al. are inflated and therefore misleading. In a recent survey among German researchers, they rephrased some of the QRPs from the John et al. survey. For instance, instead of "failing to report all of the study’s dependent measures,” they asked about "failing to report all dependent measures *that are relevant for a finding*.” Moreover, they made a distinction between different types of prevalence rates. Interestingly, the prevalence rates reported by Fiedler and Schwarz are substantially lower than those reported in the John et al. survey. Disregarding the issue of prevalence for the time being, please note that all of these research practices are labeled as "questionable” (see Table 1), because they lead to an increased chance of false positives in null-hypothesis significance testing. That is, these practices lead to an inflated chance that the null hypothesis is falsely rejected.

In an influential paper, Simmons et al. (2011) demonstrate how undisclosed flexibility in data collection and analysis allows researchers to present almost anything as significant. It is noteworthy that Simmons et al. do not use the phrase questionable research practices in their paper but refer to these as "researcher degrees of freedom.” They do not explicitly define these degrees of freedom, but note that "In the course of collecting and analyzing data, researchers have many decisions to make: Should more data be collected? Should some observations be excluded? Which conditions should be combined and which ones compared? Which control variables should be considered? Should specific measures be combined or transformed or both?” (p. 1359). These researcher degrees of freedom seem similar to the questionable research practices of John et al. (2012). However, "using degrees of freedom” does feel different from "engaging in questionable research practices.”

Recently, one of the authors played Yahtzee with his youngest son, a 10-year-old. Each time a dice would fall off the table, he wanted to re-throw the dice when the number of dots did not please him. However, he did not want to re-throw a fallen dice when the number of dots did fulfill his needs. His father explained to him that the game does not work that way because he would always win if he just kept throwing dice on the ground, deciding on the spot whether he would re-throw or not. He understood this and both decided that only dice on the table would count and all fallen dice would be re-thrown. Simmons et al. demonstrate that a similar principle holds for the hypothesis testing many of us engage in daily. Data analyses should not only count when they are significant. A priori, you will have to establish what your hypothesis is, how large your sample will be, and what types of data analyses you are going to perform. That is, in order to test your hypothesis, you will have to prespecify (see Cumming, 2014; Wagenmakers, Wetzels, Borsboom, van der Maas, & Kievit, 2012). This is called confirmatory hypothesis testing (Wagenmakers, 2012; Wagenmakers et al., 2012). Or, as Cumming seems to prefer, prespecified, question answering research. As indicated by these authors, these kinds of analyses should be distinguished from exploratory analyses (Wagenmakers et al., 2012), or question formulating data analyses (Cumming, 2014) in which data are freely explored using all of the researcher’s degrees of freedom.

The phrase "researcher’s degrees of freedom” used by Simmons et al. (2011) encompasses the fact that each researcher has many choices to make. The phrase is non-judgmental in that it seems to indicate that there are no good or wrong options here. These are choices that you make as a researcher and that you need to be aware of as much as possible. Of course, as indicated by Sijtsma, a proficient level of statistical knowledge is necessary to be able to make these choices responsibly. If this knowledge is not available, researchers should consult with methodologists or statisticians. When performing confirmatory analyses, these choices have to be prespecified. One could even argue that if analyses are not prespecified, they are exploratory by definition. However, that does not mean that non-prespecified data analyses are "questionable” or in the "gray area.” Is it a questionable research practice when you explore your data beyond your a-priori hypotheses?

The phrase "questionable research practices” seems to imply a good versus bad situation. It implies that there are "good research practices” and there are "questionable research practices.” The latter ones you should not engage in as a researcher. John et al., do indicate that there are different shades of gray. However, by labeling all of these practices as "questionable,” they all seem gray and fishy at the least.

So, how questionable are these research practices? Some of them seem beyond questionable. For instance, falsifying data and presenting them as real data is simply wrong. This can hardly be labeled as questionable. For others, however, it is questionable how questionable they are as a research practice. Some seem more questionable as a *reporting* practice than as a research practice. Please note that more than half of the practices mentioned by John et al. (see Table 1) start with "In a paper....” Take for example, the practice of "Deciding whether to collect more data after looking to see whether the results were significant.” John et al. (2012) label this as a questionable research practice. However, in itself, this is not a questionable practice (although it will inflate the chance of false positives occurring). What makes this research practice questionable is failing to report in your paper that you did this. After all, by not reporting this, the reader is unable to correctly assess the evidential value of the reported results. Moreover, if these results are reported as confirmatory, the practice is not even questionable, but plainly bad. That does not mean that researchers should withhold from exploration (by collecting more data after taking a look at the results or using any other method). They should take extreme care, however, to report explicitly what they have done, and which part was prespecified and therefore confirmatory and which part was exploratory.

## Questionable Reporting Practices

Let us engage in a small thought experiment. Imagine that, after a couple of ’failed experiments,’ a researcher decides that he knows the world better than his participants. He truly believes that the data he comes up with are more representative for the truth than the data his participants came up with. He decides to fill out a hundred questionnaires himself (at night when nobody is watching) and reports his data in a manuscript. In the method section, he clearly states that he filled out all hundred questionnaires himself, at night. Thus, he is fully transparent about the way he obtained the data.

Does this in itself constitute fraud? We do not think so. Is this bad science? We do think so. However, because this researcher has written down in his method section exactly and truthfully what he did and how he obtained the results, reviewers and editors may (and are able to) decide to what extent the conclusion the researcher draws is warranted by the research and analyses he has done. They are able to do so, because the researcher has openly and truthfully reported how his data were obtained and analyzed. Has this researcher engaged in questionable research practices such as described by John et al.? Probably not. He has not lied or left out any important information in his paper. He obviously engaged in bad science, but it is up to the reviewers and journal editors to make that call.

What makes certain research practices questionable or fraudulent are not the practices in themselves. It is the way they are reported (or not reported) that makes them questionable or fraudulent. Please note in this respect that Simmons et al. (2011) advocate six requirements for authors, five of which are about reporting: Report the rule that was used for terminating data collection; list all variables collected in a study; report all experimental conditions; report also what the results are including eliminated observations; and report results with a covariate also without the covariate. Their only requirement that does not concern a reporting issue is their advice to use at least 20 observations per cell. As noted before, Simmons et al. do not use the phrase questionable research practice in their paper. However, their requirements do seem based on their assumption that "Perhaps the most costly error is a false positive, the incorrect rejection of a null hypothesis” (Simmons et al., 2011, p. 1359).

We would like to argue that in science, there is no such thing as a questionable research practice when it concerns data analyses. Moreover, there is a danger in using this phrase because researchers that want to prevent being associated with questionable research practices or the so-called "gray area” of doing data analyses, might end up not engaging in data exploration at all. Although they might control false-positive rate, false negative rate will instead be inflated. Recently, Fiedler, Kutzner, and Krueger (2012) pointed out that there are problems with what they call a "short sighted false-positive debate.” They noted that "the failure to assertively generate and test alternative hypotheses can lead to dramatic theoretical mistakes, which cannot be corrected by any kind of rigor applied to statistical tests of the focal hypotheses” (p. 661). The cost of missing out on a new finding may very well be higher than the cost of a false positive.

In order to prevent false negatives, given all the possibilities that today’s statistical software packages offer, researchers should be encouraged to analyze, re-analyze, and re-re-analyze their data. Especially when the null hypothesis cannot be rejected with confirmatory analyses. In our experience, at least as much can be learned from analyzing a so-called ’failed experiment’ as from analyzing a study that ’worked out’ because the null hypothesis could be rejected. When all equipment worked as expected and participants participated as expected, there is no such thing as a failed experiment. When the original, theory-based, a-priori hypothesis was not confirmed, researchers owe it to themselves, to the participants that spent time on their experiment, and to the tax payer to get as much out of the data analyses as possible. However, when reporting their results, researchers should be very clear on which analyses were confirmatory and which were exploratory. The potentially (highly) questionable part of your actions as a researcher is not that you engage in all kinds of exploratory analyses. Instead, the questionable part is not reporting truthfully and explicitly the exploratory nature of these analyses. It is not the research practice that is potentially questionable; it is the reporting practice that is potentially questionable.

Pre-registration of hypotheses, sample size, and data-analysis plan should be of great benefit in this respect. We know from personal experience, how our own ideas about a study as a researcher can subtly shift during the data analysis phase. In order to be able to make a clear distinction between confirmation and exploration, pre-registration should be instrumental. In fact, one could argue that without any form of pre-registering one’s hypotheses and data analysis plan, all outcomes should be considered a result of exploration. After all, as noted by Sijtsma and many others, people fall prey easily to all kinds of information processing biases (such as hindsight, availability, confirmation, and base rate biases; see for an overview Kahneman, 2011). Also researchers unwillingly may fall prey to these biases. Pre-registration offers a helpful tool to prevent this, and we advocate using it.

After the publication of the Simmons et al. article on researcher degrees of freedom and the John et al. article on questionable research practices, many researchers and students have started questioning the way they themselves perform data analyses, and the way colleagues perform theirs. We have been involved in lab meetings where we had to explain to students that excluding outliers is not a questionable research practice in itself, given that you define your exclusion rule a priori. However, not all anomalies can be discussed in a prespecified data analysis plan. Also aposteriori, however, retaining outliers in a data set may result in a more distorted picture than removing them (for instance, when a result is driven completely by one or two outliers). We tried to explain to the students that removing outliers could be a healthy research practice, as long as you truthfully report whether you removed these data points based on a-priori exclusion rule or based on post-hoc criteria. Again, the only thing that can be questionable is the reporting, not the data analysis technique itself.

In our opinion, there is a risk in using the phrase questionable research practice to describe certain forms of data analyses. As noted by Sijtsma, exploration and exploratory analyses play a crucial role in science. When theory is unavailable, based on exploration, first theoretical ideas may be formed. Of course, ideas that result from exploration should subsequently be tested in a confirmatory way using new data. Nevertheless, many now confirmed theories have found their origin when researchers explored unexpected findings in a data set. The risk of considering certain data analysis practices as questionable is that future scientists may refrain from using these practices to prevent being associated with such questionable practices. This could be an expensive mistake that may lead to false negatives, and may hurt the further (theoretical) development of our field. Researchers should not avoid exploring to prevent being associated with "questionable” practices. On the contrary, we should stimulate exploration perhaps as much as we should stimulate truthful and transparent reporting of what we have done and when we have done it.

It should be clear by now that our suggestion is to avoid the phrase "questionable research practices” when in fact we discuss data analysis practices, of which it is questionable how questionable they are. What we should focus on is how to prevent questionable *reporting* practices. Making an explicit distinction between prespecified (ideally preregistered), confirmatory analyses and exploratory analyses is crucial in this respect. Explicating this distinction in a paper could mean the difference between a truthful reporting practice and a questionable reporting practice.

## Conclusion

Sijtsma makes an important point. Prevention of questionable research practices should be more important than detection. Moreover, we fully support his two policy measures. Whenever possible, researchers should make their research materials and research data available to colleagues for verification. Also, consulting a methodologist or statistician should be a more common practice. In general, consulting colleagues during all stages of the scientific is a great way to prevent errors.

Our additional suggestion, however, is to reserve the phrase "questionable research practices” for unethical research practices such as falsifying data, dealing with participants in unethical ways, and mistreating animals. In fact, all of these should be labeled as bad research practices. Most of the "gray area” of data analyses activities described by John et al. (2012) should be referred to as "questionable reporting practices.” Science is built for a large part on exploration, and we should avoid false negatives by focusing merely on confirmatory analyses (see Fiedler et al., 2012). At the same time, however, to avoid false positives, we should report explicitly and honestly in our papers, which analyses are prespecified and thus confirmatory and which ones are exploratory. Pre-registration provides a wonderful aid for this. In doing so, researchers may need to provide more details in their method and results sections (or use an online addendum for this). Yes, we will run the risk of bothering our readers with "tales of woe” (see Bem, 1987), lengthening our papers, or writing online addenda that may not be that easy to read for less-informed readers. However, this may be the only way to avoid false positives without the risk of obtaining too many false negatives. Exploring data should not become associated with, or become labeled as, a questionable research practice. It is how researchers, objectively, report analyses that enables one to differentiate between good and bad science.

## Footnotes

1 (https://groups.google.com/forum/#!msg/spsp-discuss/6oP7QwsfBXM/bZzsx8AOafQJ).

## References

[CR1] Bem DJ, Zanna MP, Darley JM (1987). Writing the empirical journal article. The complete academic: A practical guide for the beginning social scientist.

[CR2] Cumming, G. (2014). The new statistics: Why and how. *Psychological Science*, 7–29. doi:10.1177/0956797613504966. 10.1177/0956797613504966 24220629

[CR3] Fiedler K, Kutzner F, Krueger J (2012). The long way from alpha-control to validity proper: Problems with a shortsighted false-positive debate. Perspectives on Psychological Science.

[CR4] Fiedler, K., & Schwarz, N. (2014). Questionable research practices revisited. Unpublished.

[CR5] John LK, Loewenstein G, Prelec D (2012). Measuring the prevalence of questionable research practices with incentives for truth telling. Psychological Science.

[CR6] Kahneman D (2011). Thinking, fast and slow.

[CR7] Simmons JP, Nelson LD, Simonsohn U (2011). False-positive psychology: Undisclosed flexibility in data collection and analysis allows presenting anything as significant. Psychological Science.

[CR8] Sijtsma, K. (2015). Playing with data. Or how to discourage questionable research practices and stimulate researchers to do the right thing. doi:10.1007/s11336-015-9446-0. 10.1007/s11336-015-9446-0 25820980

[CR9] Wagenmakers E-J (2012). A year of horrors. De Psychonoom.

[CR10] Wagenmakers EJ, Wetzels R, Borsboom D, Van der Maas HLJ, Kievit RA (2012). An agenda for purely confirmatory research. Perspectives on Psychological Science.

